# Preface

**DOI:** 10.1093/jxb/erv067

**Published:** 2015-03-11

**Authors:** Katja Graumann

**Affiliations:** Oxford Brookes University, UK

Part of this special edition highlights the latest advances made in understanding the structure and functions of nuclear periphery components in plants. The plant nuclear envelope (NE), with its inner and outer nuclear membrane components, the NE–embedded nuclear pore complexes (NPC) and the NE–associated nucleoskeletal plant lamina have been of great interest in recent year. While some of their components and structures are conserved in other eukaryotes, for instance the majority of nucleoporins, many plant-specific NE associated proteins and functions have been discovered. As a result, we have gained fascinating new insights into how plant nuclear periphery components affect various nuclear functions such as gene regulation, chromatin organisation and nuclei positioning. Both Tamura *et al.* and Zhou *et al.* focus on that latter aspect, particularly the role of cytoskeletal – nucleoskeletal bridging complexes and their roles not just in nuclear position but also nuclear shape regulation. Tamura *et al*. explore in more detail the function of novel nucleoskeletal proteins specific to plants and how nuclear morphology effects plant cell function. Zhou *et al*. review the various functions of Sad1/UNC84 (SUN) domain – Klarsicht/Anc1/Syne homology (KASH) bridges with particular emphasis on plant-specific KASH proteins and their role in plant development. The impact of the nuclear periphery, and in particular of nucleoporins, on gene regulation is comprehensively examined by Parry. The author also illustrates the role of NPC and NE components in chromatin organisation and RNA export. In addition, MacGregor and Penfield detail the complex functions and pathways that are affected by the nucleoporin HOS1. This protein is a fascinating example of how nuclear periphery components affect signalling pathways during plant growth and development – in the case of HOS1 during temperature responses and flowering time. Schubert and Weisshart present novel insights into the distribution of active and inactive RNA Polymerase II molecules as revealed by super resolution microscopy techniques Structured Illumination Microscopy (SIM) and Photoactivated Localization Microscopy (PALM). Finally, Petrovska *et al*. compare various techniques used for identifying nuclear components in plants – including NE associated proteins. This is particularly exciting as still only a few membrane-intrinsic NE components are known. In addition, most of the identified NE, NPC and nucleoskeletal proteins identified still remain to be functionally characterised, such as many of the nucleoporins. The research presented here is an example of the exciting discoveries that are driving this field forward. The authors are part of the International Plant Nucleus Consortium (IPNC), which is a platform to facilitate networking and sharing of researchers and their expertise of the plant nucleus.

